# The Schools of Boston

**Published:** 1864-09

**Authors:** 


THE SCHOOLS OF BOSTON.—[The following from the *Boston Medical and Surgical Journal,* should be memorized, and "improved" by all parents. It is as applicable to the West as to the East—as important to the children of Dentists as to the offspring of others. Ed.] We have again and again expressed our opinion in relation to the system of over study pursued in our public schools, and we believe that most physicians agree with us in our condemnation of this murderous policy upheld by the city in spite of continued remonstrance. So strong was the feeling in the profession just before this all-absorbing struggle for liberty and nationality drew its attention away from home affairs, that the matter was discussed at several meetings of our District Medical Society, and the almost unanimous opinion prevailed that the present system of school instruction was ruinous to the health of the children of Boston. There is probably not a physician in the city who has not occasion every year to insist upon the removal from school of boys or girls who are breaking down of mental over-work. One of our daily journals, always most interested in matters of education, appeared with a most appropriate leader on the morning of the recent annual school jubilee, from which we give the following extract, and which we would like to present to our readers in full. We hope that the public press will not let the matter rest here, but will continue to remind our community that it is one of far greater importance than an unpleasant odor, which excites popular indignation to a white heat, and one, moreover, which should not be trifled with longer.

We can not agree with the *Advertiser* on one point, however. It exempts the School Committee from blame, so long as it "executes but the public demand." It seems to us that the Committee is almost wholly responsible for the evil. In the effort made by the Suffolk District Medical Society above alluded to, the numerous individual instances of grave disease, cited by physicians as evidence of the injurious working of the school system, were always answered by the Committee with the argument that study was forbidden out of school, and that if parents allowed their children to transgress these rules to the detriment of their health, they alone were responsible. The absurdity of such a statement is openly apparent. The Committee sets up a standard of requirements, which are and can be fulfilled only by the most strenuous mental exercise on the part of the pupils, who are stimulated by rivalry and ambition up to a pitch of intense nervous excitement as the time for the distribution of rewards approaches. We would like to know how many of the present list of medal scholars there are who have not studied at home every day for months? How can it be otherwise when probably not ten out of the graduating class at Cambridge could pass the examination required at the Normal School? No wonder that those who stand highest among its graduates leave it pale and thin. What folly, then, for the School Committee to attempt to transfer the responsibility of its own acts to the misjudged ambition of parents.

"Leaving the system, to discuss the present tendency in practice of the schools, it is evident that careful parents, who care more for the health of their children than for their laurels, more and more regularly attempt to withdraw them from the public schools. For reasons of which we shall speak. The boys escape the ordeal much more successfully than their sisters. But that is only a rare exception. When do we find any skillful physician entrusting his daughters even to the best public school? Yet there is virtually no choice. The private schools are worked at as high a pressure. Their teachers are intelligent enough to regret it, as are their fellow-laborers who work for the public, but that vitiated public sentiment or public indifference which mistakes book-learning for wisdom, drives them up to the overwork which, with very few exceptions, is the vice of our whole system.

"On the other hand, we constantly hear of children withdrawn, where the direction of the physician is the reason assigned. The strain on the whole system is so severe, just at the period of life when the physical functions should be gaining strength, that a medal or a diploma is rightly considered poor pay for epilepsy, for dyspepsia, for typhoid fever, or for pulmonary disease.

"The boys, as has been intimated, take this thing a good deal into their own hands. But girls can not 'go into water,' can not play cricket on the common, can not form drill clubs; and yet, though the earlier development of women makes it specially necessary that we should relieve them earlier than boys from school, by a sort of fatality we pile upon them a mass of additional sciences which the boys, by some good fortune, escape from. At fourteen, most of the boys throw the whole thing up. Their wages are worth something to their parents, or they themselves decline to have anything more to do with the public schools. Four or five years are left them, therefore, to renew or create physical vigor before the age of growth is over. The girls, at the same age, are at the most critical period of life. The body is growing most rapidly, its functions are undergoing the most critical changes; its organs are adapting themselves to the necessities of womanhood—and yet at that precise period it is that we say the rest of the body may look out for itself, but that what we care for is brain, and nothing but brain. The blood shall feed the brain with such nutrition as it can give, and all the rest of the system may go. Still we will not give appetite enough to endow the blood tolerably; for we will not give air or exercise enough to create a healthy appetite. We will have girls who can explain to us the binominal theorem; who can tell us how many metaphors there are in the 'Bugle Song,' and how many metacarpal bones they have. If they can do this, it is no matter, we say, whether their metacarpal bones can sustain the weight of a pail of water, or whether they themselves are ever fresh enough or free enough to have written for themselves a 'Bugle Song.'

"Now this becomes a serious matter—when, as a generation passes, we find that half our young men are exempt from bearing arms by physical weakness, and that half our young women, in what was once the prime of life, are confirmed invalids. It is a serious matter, when for the class which graduates this year at the Normal School, we find that there is another class, as large, of those who have dropped out by the way, unable to bear the high pressure of the Grammar schools and of the Normal. Such facts of themselves show that the practice is as disastrous as the system is absurd.

"The truth has been substantiated in the science of education, that growing children acquire as much in three hours' daily study as they can acquire in a day. On this truth, any true system must be founded. Beyond this period, the 'power of acquisition,' as the English teachers call it, is at an end. If, then, we keep the children in school more than for three hours of study, it is for our convenience, not for their instruction. It may be desirable to assign an hour or more for recitation beyond these three hours; but let it be remembered by all parties, that that is for the convenience of the public, or to satisfy the tax-payer's passion for paying, or to keep the homes of the parents quiet. Let no one pretend that the benefit of the scholar is involved. The true amendment, as we believe, of the folly of the last generation, which has now about run its course, is to be found in some respect for this central truth in education. Let some spirited man of sense in the School Committee move a reduction of the term of school to four hours a day. He will be met with a howl, as if he proposed to set fire to all the school houses. But let him grimly persevere, let him lighten up his argument with cheerful instances of dropsy on the brain, of sleepless nights, of early insanity, and of the rest on the dark side of that medal whose obverse bears Franklin's smile. Let him urge this steadily for a year or two, till some one proposes a compromise on five hours. Let him then begin with a proposal for three hours daily, and go through the same ordeal till we have come down to four. Four hours, three for study and one for recitations, with proper recesses, and with no evening lessons, would teach the children all that they learn now, and would give them some chance to be strong and wise as well, where now we are satisfied if they are learned.

"It is with some regret that we throw in our protest in the midst of a holiday. It is because this is the only day when the public care for the schools, that we do so. The teachers are not to blame. They see with agony the work of the system. But each of them says that his particular school must keep pace with each other school in the multitude. The School Committee are not to blame, so long as they do but execute the public demand. It is the public which must be taught, that nothing is gained by the high-pressure system; that home-quiet is dearly purchased, when school-confinement drags down the vital power of children, and that the best authorities on education in the world unite in protesting against such confinement and stimulus as are practiced in our schools."

